# Carbamazepine Alleviates Retinal and Optic Nerve Neural Degeneration in Diabetic Mice via Nerve Growth Factor-Induced PI3K/Akt/mTOR Activation

**DOI:** 10.3389/fnins.2019.01089

**Published:** 2019-11-01

**Authors:** Nehal M. Elsherbiny, Yousra Abdel-Mottaleb, Amany Y. Elkazaz, Hoda Atef, Rehab M. Lashine, Amal M. Youssef, Wessam Ezzat, Sabah H. El-Ghaiesh, Rabie E. Elshaer, Mohamed El-Shafey, Sawsan A. Zaitone

**Affiliations:** ^1^Department of Biochemistry, Faculty of Pharmacy, Mansoura University, Mansoura, Egypt; ^2^Department of Pharmaceutical Chemistry, Faculty of Pharmacy, University of Tabuk, Tabuk, Saudi Arabia; ^3^Department of Pharmacology and Toxicology and Biochemistry, Faculty of Pharmaceutical Sciences and Pharmaceutical Industries, Future University in Egypt, Cairo, Egypt; ^4^Biochemistry and Molecular Biology Department, Faculty of Medicine, Suez Canal University, Ismailia, Egypt; ^5^Biochemistry and Molecular Biology Department, Faculty of Medicine, Portsaid University, Port Said, Egypt; ^6^Department of Histology and Cytology, Faculty of Medicine, Mansoura University, Mansoura, Egypt; ^7^Department of Clinical Pharmacology, Faculty of Medicine, Suez Canal University, Ismailia, Egypt; ^8^Department of Physiology, Faculty of Medicine, Taibah University, Medina, Saudi Arabia; ^9^Department of Physiology, Faculty of Medicine, Suez Canal University, Ismailia, Egypt; ^10^Department of Physiology, Faculty of Medicine, Ain-Shams University, Cairo, Egypt; ^11^Department of Pharmacology, Faculty of Medicine, Tanta University, Tanta, Egypt; ^12^Department of Pharmacology, Faculty of Medicine, University of Tabuk, Tabuk, Saudi Arabia; ^13^Pathology Department, Faculty of Medicine (Boys), Al-Azhar University, Cairo, Egypt; ^14^Department of Anatomy and Embryology, Faculty of Medicine, Mansoura University, Mansoura, Egypt; ^15^Physiological Sciences Department, Fakeeh College for Medical Sciences, Jeddah, Saudi Arabia; ^16^Department of Pharmacology and Toxicology, Faculty of Pharmacy, Suez Canal University, Ismailia, Egypt; ^17^Department of Pharmacology and Toxicology, Faculty of Pharmacy, University of Tabuk, Tabuk, Saudi Arabia

**Keywords:** carbamazepine, mouse diabetic retinopathy, NGF, PI3K/Akt/mTOR pathway, neuroprotection, apoptosis

## Abstract

**Aim:** Diabetic retinopathy causes loss of vision in adults at working-age. Few therapeutic options are available for treatment of diabetic retinopathy. Carbamazepine (CARB), a widely used antiepileptic drug, was recently accounted for its neuroprotective effect. Nerve growth factor (NGF) activates various cascades among which, PI3K/Akt/mTOR pathway has a vital action in NGF-mediated neuronal differentiation and survival. This study evaluated the effect of CARB in the treatment of diabetic retina and unveiled some of the underlying molecular mechanisms.

**Main Methods:** Alloxan diabetes model was induced in 36 albino well-acclimatized mice. After establishment of the diabetic model in 9 weeks, mice were assigned to treatment groups: (1) saline, (2) alloxan-diabetic, (3 and 4) alloxan+CARB (25 or 50 mg per kg p.o) for 4 weeks. After completion of the therapeutic period, mice were sacrificed and eyeballs were enucleated. Retinal levels of NGF and PI3K/Akt were assessed using real-time polymerase chain reaction. Further, total and phosphorylated TrKA, PI3K, Akt, mTOR as well as Caspase-3 were measured by Western blot analysis.

**Key Findings:** Histopathological examination demonstrated that CARB attenuated vacuolization and restored normal thickness and organization of retinal cell layers. In addition, CARB increased pTrKA/TrKA ratio and ameliorated diabetes-induced reduction of NGF mRNA and immunostaining in retina. Additionally, it augmented the mRNA expression of PI3K and Akt, as well as the protein level of the phosphorylated PI3/Akt/mTOR.

**Significance:** Results highlighted, for the first time, the neuronal protective effect for CARB in diabetic retina, which is mediated, at least in part, by activation of the NGF/PI3K/Akt/mTOR pathway.

## Introduction

The increased prevalence of diabetes is gaining a great concern worldwide. The International Diabetes Federation expected that the global number of diabetic patients will rise from 285 million diabetic patients in 2010 to 552 million by 2030 (Zochodne and Malik, 2014). Epidemiological studies have shown that more than one-third of diabetic patients worldwide suffer from diabetic retinopathy and approximately 1 in 10 suffer from high levels of diabetic retinopathy that threaten the vision (Yau et al., 2012).

The main treatment options are laser photocoagulation, ocular surgery and anti-vascular endothelial growth factor (anti-VEGF) for vision-threatening diabetic retinopathy (Wong and Sabanayagam, 2019). Some studies have been conducted on the use of statins and fibrates as a supplementary treatment option to delay the progress of the disease (Shi et al., 2018).

Substantial progress has been made to reduce blindness due to diabetic retinopathy in high-income countries, such as orchestrated efforts directed toward public health education leading to early detection and the availability of effective therapeutic options (Wong and Sabanayagam, 2019). Therefore, the search for newer, less invasive, as well as inexpensive treatment options is imperative.

Insulin resistance and prolonged hyperglycemia are of the main reasons behind blood retinal barrier dysfunction and diabetic retinal damage (Mysona et al., 2014). Neurodegeneration is a crucial factor in early diabetic retinopathy. Diabetic retinopathy harms the optic nerve by producing damage to its integrity and conductivity (Victor, 2018).

Different neuronal cells, including ganglion and amacrine cells, suffer pathogenic alterations (Piano et al., 2016) manifested as neuronal apoptosis and morphologic abnormalities (Ali et al., 2019). Indeed, accumulating evidence suggested that vascular changes are preceded by retinal neuronal death, which suggests a potential therapeutic approach using neuroprotective agents (Piano et al., 2016).

Recently, neurotrophins have been documented to have an evolving role in diabetic retinopathy. Neurotrophins, such as NGF among many others, are vital for growth and survival of retinal neurons. Initially, neurotrophins are synthesized in a pro-form and then go through proteolytic cleavage for generation of the mature form that stimulates the tyrosine kinase tropomycin receptor (Trk) (Cunha et al., 2009). Thereafter, NGF binds to its respective TrkA to initiate various signaling pathways, such as PI3 kinase, Akt 1, Ras/Extracellular signal-regulated kinases (ERK), protein kinase C (PKC), as well as mammalian target of rapamycin (mTOR) pathways. This promotes neuronal cell growth, differentiation and survival (Miyamoto and Ogura, 1999; Hammes et al., 2011). Therefore, targeting these events could be useful neuroprotective therapeutic strategies for the prevention of diabetic retinopathy development.

The use of antiepileptic drugs in non-epilepsy disorders is a well-established trend. Antiepileptics are clinically important in the treatment of many neurologic disorders, such as migraine, neuropathy and some psychiatric diseases (Johannessen, 2008). The pharmacological action of different antiepileptic drugs is directed toward control over the disturbance in neuronal excitability through modulation in ion channels, receptors, cell expression or signaling pathways (Johannessen, 2008; Reda et al., 2016). Additionally, antiepileptic drugs, such as lamotrigine and pregabalin, cause alteration of NGF level (Johannessen, 2008; Zhang et al., 2014; Al-Massri et al., 2019).

Carbamazepine is widely used as a highly effective antiepileptic drug, exerting its action via blocking sodium channel conductance and reducing neuronal excitability. It seems interesting to explore this pathway using CARB. Moreover, CARB has been studied as a neuroprotective drug *in vivo* (Cunha et al., 2009; Woronowicz et al., 2012) and *in vitro* (Bown et al., 2003; Rekling, 2003). However, the possible neuroprotective action for CARB in diabetic retinopathy has not been reported.

Since retinal NGF was reported to promote neuronal survival in diabetic retinopathy and since CARB was documented as a neuroprotective agent in many neurologic disorders, this study explored the possible retinal protective action of CARB in diabetic mice. The aim of this study is to test the effect of CARB in alloxan-induced diabetic mice on retinal NGF and pTrKA/TrKA ratio, as well as possible modulation of PI3K/Akt/mTOR pathway.

## Materials and Methods

### Animals

Thirty-six male Swiss albino mice [body weight range equals 25–30 g] were randomly selected to be used in the experiment. Animals were purchased from Moustafa Rashed Company for Laboratory Animals (Cairo, Egypt). The mice were 12 weeks old at the beginning of the experiment. They were kept in clean plastic cages in a normal day/night cycle and temperature equals 25 ± 5°C with food and water *ad libitum*. The experiment was performed after permission (No. 201603A2) from the Research Ethics Committee at Faculty of Pharmacy, Suez Canal University in agreement with the principles of the Basel Declaration.

### Selection of Protective Doses

A previous experimental study in mice selected 200 mg/kg as a daily therapeutic dose for CARB up to 2 months. Authors did not notice a major adverse effect for this dose throughout the course of treatment (Zhang et al., 2018). In one study, mice were given a 50-mg/kg dose of CARB orally, daily for a couple of weeks (Chen and Ma, 2017). Another study tested the anticonvulsant effect of oral CARB in rats at 35 and 50 mg/kg (Dhande et al., 2009). Before the start of this study, a preliminary experiment was performed, in which mice were given 25 and 50 mg/kg CARB orally on 48 h basis; this schedule was fine and provided a promising retinoprotective effect, hence was used in the current study.

### Experimental Design

Following acclimatization for 1 week, mice were assigned to 4 groups: (i) saline control, (ii) alloxan diabetic, (iii) alloxan+CARB (25 mg/kg), and (iv) alloxan+CARB (50 mg/kg) groups. Type 1 diabetes mellitus was prompted through one subcutaneous injection of alloxan hydrate (dissolved in saline, 180 mg/kg, SD fine-chem limited, Mumbai, India). However, the saline control group administered an injection of saline (10 ml/kg). Mice were counted diabetic when the glucose level in blood was >250 mg/dl 1 week after injection of alloxan. After 9 weeks, mice in group (iii) and (iv) received oral CARB (25 and 50 mg/kg, every 48 h) for 4 weeks. The diabetic control group was given 2% solution of carboxymethylcellulose in distilled water (vehicle of CARB).

Under ketamine HCl anesthesia (100 mg/kg, i.p.), animals were sacrificed using cervical dislocation. Eyeballs were enucleated and the left one was fixed with 1% paraformaldehyde solution, and then processed for histological staining at the level of the retina. The right dissected retinas were divided into 2 halves and then maintained at −80°C until further qRT-PCR and Western blotting studies were completed.

### Assessment of Retinal NGF, PI3K, and Akt Gene Expression

RNeasy mini kit [Qiagen] was used for extracting total RNA from retinal tissue following the manufacturer's procedure. The quality and quantity of extracted RNA in each sample was then evaluated using NanoDrop ND-1000 spectrophotometer [NanoDrop Tech., USA] in each sample. Reverse transcription was done for the extracted RNA to cDNA using a high capacity cDNA reverse transcription kit commercially available by Applied Biosystems [P/N4368814]. Mastercycler Gradient Thermocycler from Eppendorf [Germany] was used for reverse transcription at 25°C (10 min), then 37°C (2 h) followed by 85°C (5 min), and finally held at 4°C. Genomic DNA contamination was excluded in each experiment using a control for the template and another one for reverse transcriptase.

For quantification of NGF, PI3K and Akt genes, qRT-PCR technique was applied. Taqman Universal PCR master mix II, No UNG (2×) [Applied Biosystems] and Taqman assays [Applied Biosystems], assay ID [Mm01282781_m1 for PIK3R1 (Chen et al., 2012; Elshaer et al., 2019) Mm01331626_m1 for Akt1, Mm00443039_m1 for NGF, and Mm99999915_g1 for GAPDH, the endogenous control gene] were utilized (Kanwar and Kowluru, 2009; Qiu et al., 2017) to run reactions in triplicate.

Appropriate negative and positive controls were involved in the reaction. Final volumes for PCR reactions equal 20 μl, including 1 μl 1× TaqMan® assay, 10 μl 2× TaqMan Universal PCR Master Mix, 1.5 μl cDNA, and 7.5 μl of nuclease free water using StepOnePlus Real Time-PCR system [Applied BioSystems] to perform PCR analysis. The thermal cycling conditions: 95°C for 10 min, 40 cycles at 95°C for 15 s and cycles at 60°C for 1 min.

### Western Blot Analysis for the Selected Proteins

Isolated retinal tissue was homogenized in RIPA buffer with inhibitors of protease and phosphatase. To remove insoluble material, homogenates were centrifuged at 14,000 × g at 4°C for 20 minutes. The supernatant was transferred to a new microcentrifuge tube and 5 μL was used for the determination of protein concentration using Bio-Rad Quick Start™ Bradford Protein Assay kit. Similar amounts of protein in retinal homogenate were loaded on sodium dodecyl sulfate-polyacrylamide gel after initial denaturation step using 4x Laemmli Sample Buffer (Bio-Rad, USA). Following protein separation by electrophoresis, the gels' protein was transferred to nitrocellulose membranes. For blocking of the free sites on the membranes, incubation in 5% non-fat dried milk (Bio-Rad, USA) was done for 1 h. This was followed by washing of the blocked membranes and incubation with primary antibodies for TrkA (Abcam, ab76291), phospho-TrkA (tyrosine 496, Abcam, ab111606), PI3K (ab86714), phospho-PI3K (tyrosine 607, ab182651), Akt (ab179463), phospho-Akt (serine 473, ab81283), mTOR (ab2732), phospho-mTOR (serine 2448, ab109268), and cleaved caspase 3 (ab2302) at 4°C overnight with gentle agitation. Thereafter, the blots were washed and incubated with appropriate horseradish peroxidase (HRP)–conjugated secondary antibody (goat anti-rabbit IgG H&L (HRP, ab6721) and goat anti-mouse IgG H&L (HRP, ab205719), and then the protein was visualized via enhanced chemiluminescence using the enhanced chemiluminescence ECL Advance™ Western blotting detection kit (Amersham BioSciences, Buckinghamshire, UK). The intensity of immunoreactivity was quantified by densitometry using ImageJ software (NIH).

### Histopathological Analysis and Immunohistochemistry

After completion of the treatment, mice were sacrificed, and after removal of the globe and part of optic nerve from orbit, it was fixed in formalin (150–300 ml of 10% neutral buffered formalin) for 24 h before grossing and without opening or puncturing the eye. It was then washed in running tap water for 30 min and placed in 60% ethyl alcohol for 2 h. The orientation of the globe was determined based on dimension of the cornea, optic nerve and insertion of the oblique muscles then the eye was cut open using a sharp razor. Each eye was then opened with a cutting motion from back to front. The plane of each section begins close to the optic nerve while ends through the peripheral part of the cornea. The globe was cut along a horizontal plane. After examination of the interior of the globe and after placing the eye flat on its cut surface, a second plane of section was obtained, parallel to the first, again passing from back to front.

To prepare cross sections of tissues, serials of different ethyl alcohol concentrations (70, 80, 95, and 100%, respectively) were used. The serial of xylol solution and the eyes were embedded in paraffin, and then they were cut into 4–5 μm sections and stained with hematoxylin and eosin (H+E) or NGF immunohistochemistry. Finally, they were mounted; cover slipped and evaluated for morphopathological changes in the retina of diabetes and treatment groups.

For immunohistochemistry, deparaffinized sections were placed on slides coated with poly-L-lysine. The procedure of immunostaining was completed according to a previous method (Taylor, 1994). Sections were immersed in citrate buffer (0.01 M, pH 6.0) in a microwave for 15 min for routine antigen retrieval. Endogenous peroxidase activity was blocked by 3% hydrogen peroxide for 15 min and sections were incubated with primary rabbit monoclonal antibody to NGF beta (Catalog#: EP1320Y, BioGenex). A chromogen (3,3′-Diaminobenzidine (DAB) was then applied for 1 min. Counterstaining of the sections was done using Mayer's hematoxylin for 3 min. Primary antibodies were omitted to obtain negative controls, and since external positive staining controls were performed in parallel with paraffin sections of cervical carcinoma.

### Retinal Examination and Measurements

Slide examination was done using light microscopy and examined under Olympus CX31 light microscope. Pictures were obtained using a PC-driven digital camera (Olympus E-620).

### Digital Image Analysis for Quantification of Immunohistochemistry

For quantification of the immunostaining, slides were visualized by an Olympus® microscope and imaged using Olympus® digital camera with 0.5× photo adaptor, using 40× objective under the same light intensity, and saved as TIFF. The images were analyzed with a specific built-in routine for object counting and analysis using ImageJ software. Two sections were prepared from each mouse, 5 random fields were analyzed from each section from each individual animal and the average was taken: the group average was used for comparison. Briefly, employing the color deconvolution plugin, the area stained with DAB was alienated from hematoxylin, which converts the color information to red, green, and blue (RGB) images with multiple stains (Ruifrok and Johnston, 2001). This step was done by calculation of the involvement of every stain according to stain specific RGB absorption. After that, images were changed to 8-bit type and treated into binary color image. Calibration of the measuring icon assisted determination of the % of immunostained area, which lastly appeared as a black color.

### Statistical Analysis

The fold change of mRNA expression in samples was relatively compared with the mean of control samples using the equation (2^−ddCT^) based on the threshold cycle. Data from PCR were demonstrated as box-plots and analyzed by means of non-parametric Kruskal-Wallis test followed by Mann-Whitney U test. However, quantitative data from immunohistochemistry showed normal distribution and was demonstrated as mean ± SD. The judging of the statistical difference between the data from the different groups was done by the one-way analysis of variance test and Bonferroni's *post-hoc* test at *P* < 0.05.

## Results

### Establishment of Alloxan-Induced Diabetes Model

In the present work, Alloxan-treated mice showing fasting glucose level that exceeded 250 mg/dl were selected. After completion of the therapeutic regimen, fasting blood glucose in the different groups was as follows: vehicle group: 92.67 ± 8.5 mg/dl, alloxan group: 401.17 ± 111.3 mg/dl, alloxan+CARB (25 mg/kg): 375.17 ± 121.69 mg/dl and alloxan+CARB (50 mg/kg): 393.83 ± 119.83 mg/dl. Statistical analysis revealed noteworthy differences between the last three groups vs. the saline group. However, there was no significant difference between the mice groups that received CARB vs. the alloxan control group (data are not shown in illustrations).

Mortality percent in experimental groups was determined. The saline group showed 11.11% mortality (8 mice survived), while alloxan-diabetic group showed 33.33% mortality (6 mice survived). Further, the alloxan+CARB (25 mg/kg) group showed 22.22% mortality (7 mice survived) and alloxan+CARB (50 mg/kg) group showed 33.33% mortality (6 mice survived) (data not shown in illustrations). The difference between the study groups did not reach statistical significance. For performing different assays, 6 mice from each group were used.

### Histopathological Examination of Retinal and Optic Nerve Sections

**Figure 1:**
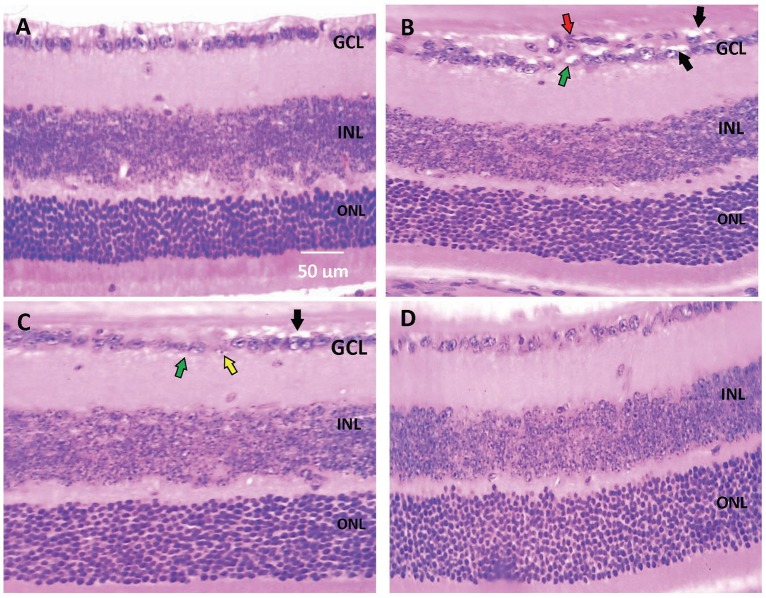
Photomicrograph for hematoxylin and eosin stained retinal sections. **(A)** Saline group showing normal well-arranged retinal layers. Ganglion cell layer (GCL); Inner nuclear layer (INL); Outer nuclear layer (ONL). **(B)** Alloxan diabetic group showing disorganization of ganglion cell layers (red arrow), vacuolar degeneration of most of the ganglion cells (black arrows) and marked edema (green arrows). **(C)** Alloxan+CARB (25 mg/kg) group showing mild vacuolar degeneration of most of few ganglion cells (black arrows), ballooning degeneration with pyknotic nuclei (yellow arrow) and mild edema (green arrow). **(D)** Alloxan+CARB (50 mg/kg) group showing restored thickness of organized retinal cell layers with significant attenuation of vacuolization's and neovascularizations (H&E, ×400). CARB, carbamazepine.

Histopathological examination of retinal sections (*n* = 6 in each group) stained with H+E indicated that retinal layers in the saline group were well-arranged. Intact and organized layers from the top to bottom of the section; ganglion cell layer (GCL), inner plexiform layer (IPL), inner nuclear layer (INL), outer plexiform layer (OPL), and outer nuclear layer (ONL). However, retinas from the diabetic group demonstrated pathologic abnormalities with distorted organization of cell layers with prominent edema, vacuolization and some vessel leakage. Diabetic animals that received CARB (25 mg/kg) showed well-organized retinal cell layers with minimal vacuolization. Furthermore, diabetic animals that received CARB (50 mg/kg) group showed significant suppression of vacuolization, in addition to restoration of the organization of retinal cell layers as shown in Figures 1A–D.

**Figure 2:**
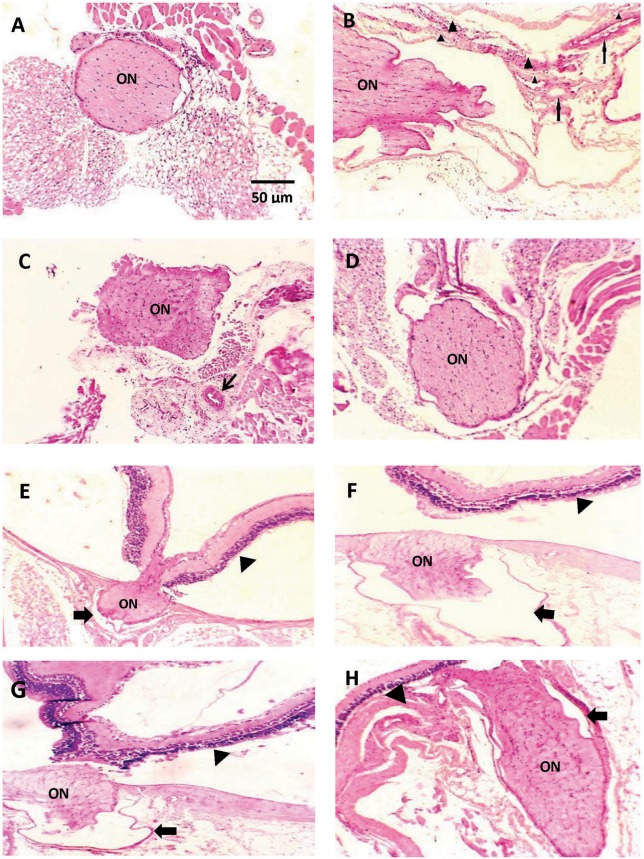
Photomicrograph of histological sections of optic nerve stained with hematoxylin and eosin. Sections are shown in **(A)** image from saline group shows meningeal sheath closely intact to the optic nerve, **(B)** image from diabetic group showing optic nerve with atrophic changes, papillary projections in wide subarachnoid space, thickened wall of blood vessels is noted (thin arrows) with fibrous deposition (arrow head) **(C)** image from alloxan+CARB (25 mg/kg) treated group showing less widening of subarachnoid space and thickened blood vessel wall (thin arrow), **(D)** image from alloxan+CARB (50 mg/kg) treated group showing significant attenuation of diabetic changes and nearly normal structure of optic nerve (H&E, ×400). **(E)** Image from saline group shows, thin-walled pia with regular meningeal sheath closely intact to the optic nerve and retina, **(F)** image from diabetic group, optic nerve with atrophic changes, wide subarachnoid space and complete detachment from retina, the nerve sheath appears distorted (thick arrow), **(G)** image from alloxan + CARB (25 mg/kg) treated group, mild separation from retina and its sheath which shows irregularity, **(H)** image from alloxan + CARB (50 mg/kg) treated group, optic nerve attached to the retina with closely intact regular meningeal sheath (H&E, ×400). Thick arrow, meningeal sheath; arrow head, retina; ON, optic nerve.

Figures 2A–D demonstrates a photomicrograph of cross section of optic nerve stained with H+E. Saline control group showed meningeal sheath closely intact to the optic nerve, whereas the diabetic group showed atrophic changes in the optic nerve with papillary projections in wide subarachnoid space. Furthermore, the wall of blood vessels was thick with fibrous deposition. Nerves from mice that received CARB (25 mg/kg) showed less widening of subarachnoid space and thickened blood vessel wall, meanwhile, CARB (50 mg/kg) showed significant attenuation of the observed pathologic changes and nearly normal structure of optic nerve.

Figures 2E–H represents a photomicrograph of longitudinal sections. A nerve from saline control group appeared with thin-walled pia with meningeal sheath closely intact to the optic nerve and retina. The section from the alloxan-diabetic group show prominent atrophic changes and wide subarachnoid space. Optic nerves from mice that received CARB (25 mg/kg) showed mild detachment from the retina with less subarachnoid space. In addition, mice treated with CARB (50 mg/kg) displayed restored attachment to the retina with closely intact meningeal sheath.

### Expression of NGF and PI3K/Akt/mTOR

**Figure 3:**
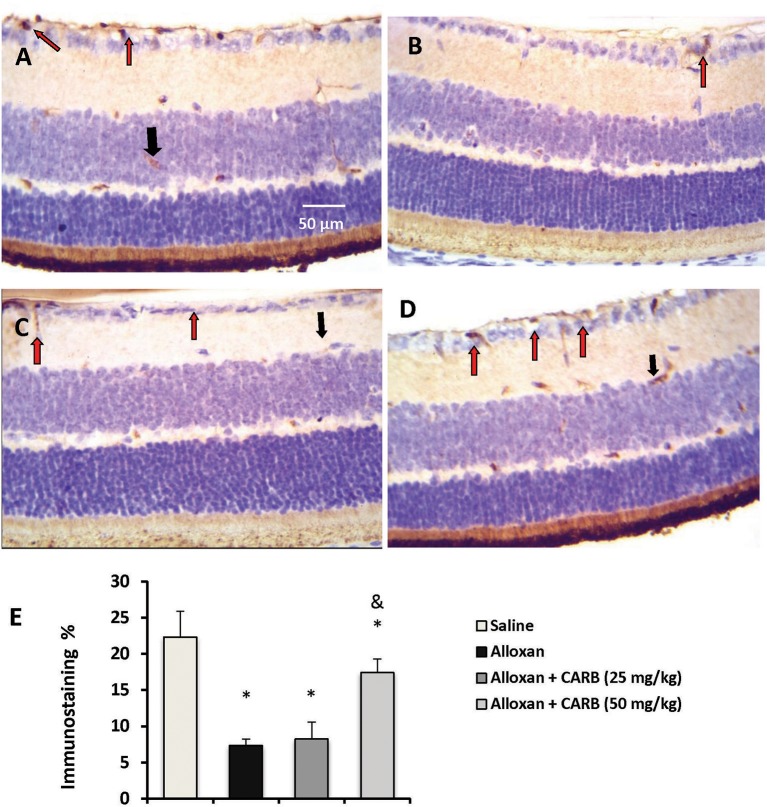
Photomicrograph for nerve growth factor stained retinal sections. **(A)** Image from saline group showing high staining in retinal ganglion cells (red arrow) and INL (black arrow). **(B)** Image from alloxan diabetic group showing relatively low staining (red arrow). **(C,D)** Images from alloxan+CARB (25 or 50 mg/kg) groups showing relatively higher staining than alloxan control group in both retinal ganglion cells and INL (red arrows and black arrows, respectively). **(E)** Column chart representing the mean area for immunostaining %. CARB, carbamazepine. Data are mean ± SD and analysis was performed applying the one-way ANOVA followed by Bonferroni's test at *P* < 0.05. *Different from saline group, ^&^Different from alloxan group.

Figures 3A–D shows retinal immunostaining for NGF. A lower degree of staining was detected in the alloxan-diabetic group. However, treatment with CARB (50 mg/kg) significantly enhanced the retinal NGF level vs. the alloxan-diabetic group (Figure 3E).

**Figure 4:**
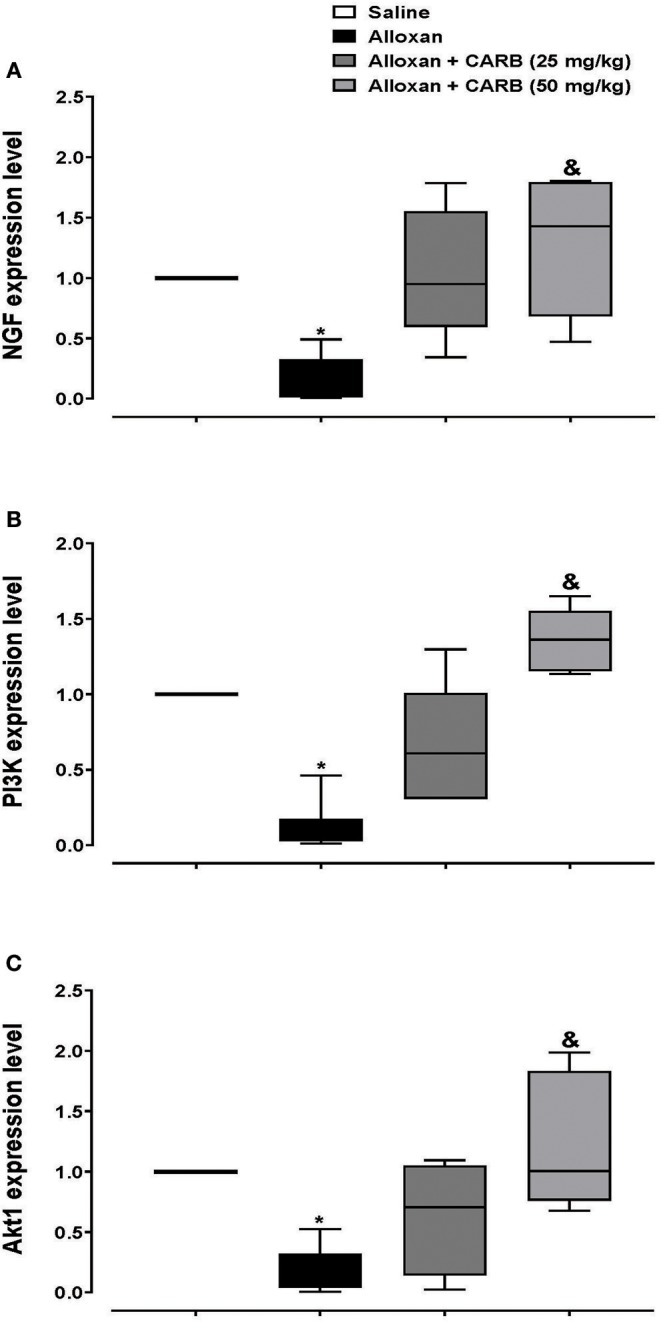
Polymerase chain reaction assay for retinal expression of NGF, PI3K and Akt genes. Data are box plots representing median and quartiles for relative expression of **(A)** NGF, **(B)** PI3K and **(C)** Akt. CARB: carbamazepine. Data are medians and quartiles and analyzed by Kruskal-Wallis ANOVA test at *P* < 0.05. *Different from saline group, ^&^Different from alloxan group.

Further, retinal mRNA expression of NGF was downregulated in diabetic mice, whereas treatment with CARB (50 mg/kg) significantly increased the retinal NGF level vs. the alloxan control group (Figure 4A). Furthermore, expression of genes encoding PI3K and Akt was lower in alloxan diabetic mice vs. saline control mice (Figures 4B,C). CARB (50 mg/kg) increased the retinal mRNA expression of these genes.

**Figure 5:**
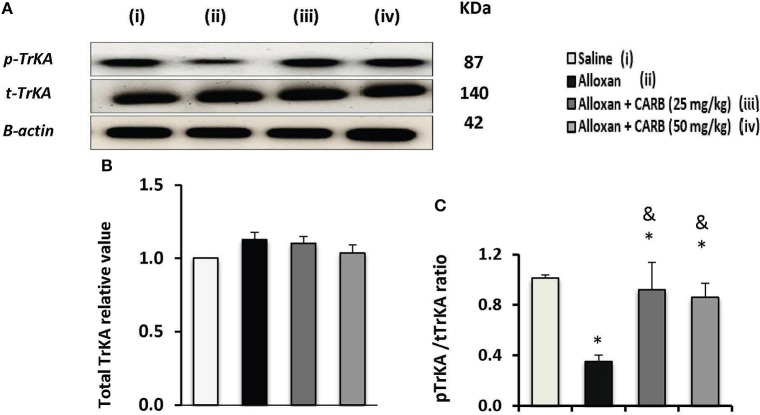
Effect of carbamazepine on retinal pTrKA and tTrKA in alloxan diabetic mice. **(A)** Western blot analysis was used for measuring the proteins of pTrKA and β-actin was used to confirm equal loading, and the intensity of immunoreactivity was quantified by densitometry. Value of each sample was normalized to the value of β-actin and averaged. The final comparison was done using the relative value to normal protein content. pTrKA: phosphorylated TrKA and tTrKA: total TrKA. Column charts representative to mean ± SD for band densities for total TrKA relative values **(B)** and pTrKA/tTrKA ratios in experimental groups **(C)**. Analysis was performed using one-way ANOVA and Bonferroni's test. *vs. saline group, ^&^vs. alloxan group. Whole Western blot runs are available in the Supplementary Materials.

Figure 5A shows Western blot analysis for total TrKA (t-TrKA) and phosphorylated TrkA (p-TrKA). Results indicated non-significant differences in tTrKA among the study groups (Figure 5B). However, the ratio of p-TrKA to the total amount was significantly lower in alloxan diabetic group. CARB (25 or 50 mg/kg) upregulated the amount of p-TrKA to a significant level and, hence, increased the calculated ratio p-TrKA/t-TrKA vs. the alloxan control group (Figure 5C).

**Figure 6:**
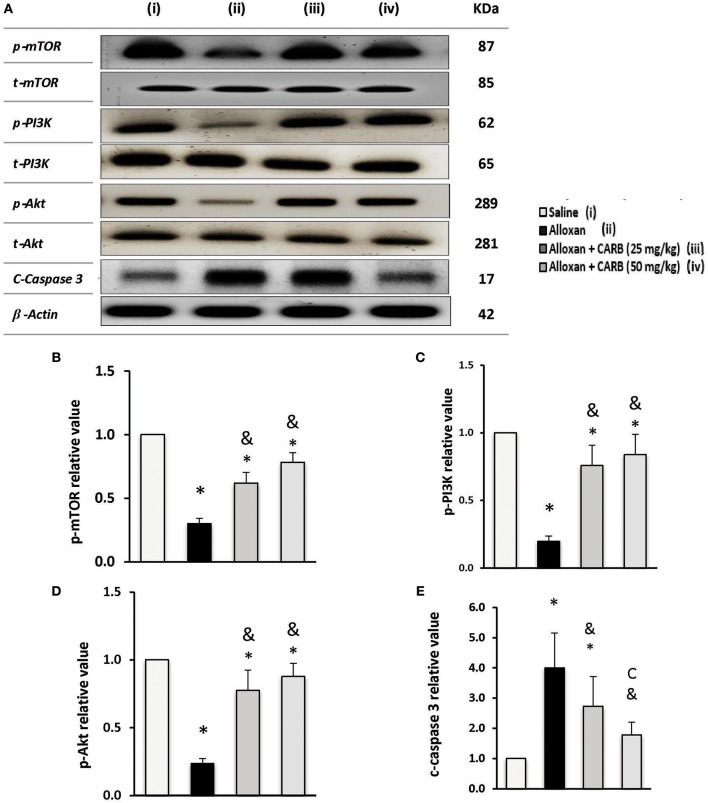
Effect of carbamazepine on retinal PI3K, Akt, mTOR and c-caspase 3 in alloxan diabetic mice. **(A)** Western blot analysis was used for measuring the proteins and β-actin was used to confirm equal loading, and the intensity of immunoreactivity was quantified by densitometry. t, total; p, phosphorylated form from the proteins. Value of each sample was normalized to the value of β-actin and averaged. The final comparison was done using the relative value to normal protein content. Column charts representative to mean mean ± SD for band densities for p-mTOR/t-mTOR **(B)**, p-PI3K/t-PI3K **(C)**, p-Akt/t-Akt **(D)** and relative value for c-caspase-3 in experimental groups **(E)**. Analysis was performed using one-way ANOVA and Bonferroni's test. *vs. saline group, ^&^vs. alloxan group. Whole Western blot runs are available in the Supplementary Materials.

Figure 6A illustrates phosphorylated and total proteins for PI3K/Akt/mTOR in the mice retinas. Low ratios of phosphorylated proteins were found in alloxan diabetic group that increased in mice groups treated with CARB (25 or 50 mg/kg) (Figures 6B–D). In contrast, retinal c-caspase 3 level was greater (4.4-fold increase) in the alloxan diabetic group (Figure 6E). Treatment with CARB dose-dependently reduced caspase 3 in retinal tissues.

## Discussion

The increased incidence and deleterious social and economic impacts of diabetic retinopathy worldwide, mandates the searching for newer, less invasive and cost-effective protective and therapeutic strategies (Chen and Ma, 2017). The current work examined the role of CARB against diabetic-induced retinal injury focusing on its influence on NGF.

In the current experiment, the alloxan model of diabetes model was employed to mimic diabetic retinopathy. This model is a well-established model that has been previously reported to induce diabetic retinopathy in mice. In a previous study, pathological changes manifested as an increase in retinal neovascularization, VEGF-A level and retinopathy score, 9 times compared to the control mice (Zhang et al., 2013). Another study indicated that alloxan-diabetic mice showed pathology in retinal GCL with vacuolization (Zhang et al., 2013). Most recently, Mohammad et al. (2019) reported that retinas in alloxan-diabetic mice showed disorganization in retinal layers, degeneration, and reduced count of retinal ganglion cells (RGCs). In agreement, another study documented that semi-thin sections of diabetic retinas in alloxan model showed disorganized retinal layers, decreased cell density in the ONL, decreased thickness and presence of pyknotic nuclei in the INL. In addition, congestion appeared in many blood vessels and the GCL contained many vacuoles (Bahr et al., 2019). Additionally, to ensure the stability of this model, blood glucose of mice in all groups was monitored throughout the experiment.

Most research assumes that neurodegeneration in the retina takes place secondarily to microvascular damage, however a growing body of evidence shows that neuroretinal alterations precede the vascular changes, and exist although clinically detectable retinal vasculopathy is absent (Yau et al., 2012; Rao et al., 2015). It was also reported in diabetic patients that neuronal death in the retina leads to a significant lessening in its nerve fibers thickness (Lopes de Faria et al., 2002). Herein, we found that diabetes affects retinal morphology and cellular integrity as depicted by reduction of thickness of nuclear layers.

Treatment with CARB restored normal thickness and organization of retinal cell layers. To explain the therapeutic effect of CARB in treating diabetic retinopathy, we focused on agents with a pivotal role in neuronal proliferation, maturation and survival, such as the neurotrophic agent NGF (Kimura et al., 2016). NGF has been reported as an anti-apoptotic molecule in various neurons, including spinal cord neurons (Lu et al., 2014) and hippocampal neurons (Nguyen et al., 2010). Indeed, RGCs and glial cells produce and utilize NGF (Mysona et al., 2014).

Beside its function in retinal neuronal developments, NGF was reported as a regulator of neuronal regeneration during retinal injury (Bartheld, 1998). Indeed, evidence from literature showed a decrease in NGF levels in serum, vitreous body and aqueous humor fluid of diabetic individuals and animals (Ali et al., 2011; Mysona et al., 2015). Therefore, restoration of the level of this protective molecule could be beneficial for inhibition of diabetic retinopathy development via prevention of neuronal cell loss. These cells are responsible for efficient communication between the eye and brain and progressive loss of these cells is a characteristic early pathogenic event in diabetic retinopathy. In the present model of alloxan-induced diabetes, there was a downregulation of retinal NGF expression and a decline in the protein level. Mounting evidence has shown that hyperglycemia induces downregulation of NGF, which may have a central contributing or initiating role in neuronal apoptosis and dropout of capillaries (Simo and Hernandez, 2015).

Herein, our results have shown that CARB treatment reversed diabetic-induced reduction of NGF mRNA and protein levels in diabetic retina. This is in accordance with recent studies showing that antiepileptic drugs exert neuroprotective effects by altering the expression of NGF and other neurotrophins (Tekgul et al., 2019). NGF can stimulate TrkA receptors. Of note, TrkA is expressed in RGCs, where TrkA activation is linked to neuronal survival (Cunha et al., 2009; Hernández and Simó, 2012; Hernández et al., 2016; Piano et al., 2016). Furthermore, activation of TrkA was linked to retinal neuroprotective effects in a model of retinal degeneration (Woronowicz et al., 2012). Interestingly, TrkA activation by selective TrkA agonists, not NGF alone, were proven to possess neuroprotective effects in various experimental models of ocular diseases, including an optic nerve injury model and rat model of high intraocular pressure (Bown et al., 2003; Rekling, 2003; Woronowicz et al., 2012). Based on these studies, it is suggested that selective TrkA stimulation is therapeutically significant in retinal neurodegeneration.

Additionally, NGF administration via ocular application in a rodent model of elevated intraocular pressure showed marked reduction in progressive RGCs loss, and excitingly, administration in patients with advanced glaucoma caused progressive enhancement in the function of inner retinal layer and conductivity (Moran et al., 2016; Chen and Ma, 2017). This was evident from enhancements in the visual field, contrast sensitivity, function of optic nerve and visual acuity; although the number of patients was small, these findings remain impressive (Lopes de Faria et al., 2002; D'Amico, 2010; Kimura et al., 2016; Moran et al., 2016).

PI3K/Akt pathway undergoes a critical role in NGF-induced neuronal differentiation and survival (Brunet et al., 2001). Interestingly, it was recently reported that NGF treatment reversed apoptosis in retinal ganglion cells via activation of PI3K/Akt (Yan et al., 2016). PI3K/Akt plays fundamental function in cell cycle regulation, growth, metabolism and survival. Activation of the PI3K/Akt pathway was considered neuroprotective in various models of neuronal toxicity (Huang et al., 2012; Yu et al., 2017), Alzheimer's disease (Ali and Kim, 2015) and cerebral ischemia (Simão et al., 2012). In the retina, activation of PI3K/Akt was protective against retinal apoptotic changes of the ganglion cells (Song et al., 2016), retinal pigment epithelial cells (Yan et al., 2014) and retinal pericytes (Haribalaganesh et al., 2009).

Further, PI3K/Akt pathway was suppressed in diabetic neurons and activation of this pathway attenuated hyperglycemia-induced myenteric neuronal apoptosis (Anitha et al., 2006). Interestingly, NGF was found to be neuroprotective against alcohol-Induced neurotoxicity through PI3K/Akt pathway activation (Liu et al., 2017; Yang et al., 2017). Similarly, we found that CARB treatment increased mRNA expression of both PI3K and Akt in the retina, suggesting that CARB attenuated diabetic-induced neuronal loss via activation of NGF/PI3K/Akt. Additionally, one *in vitro* study documented that insulin like growth factor-1 signaling, through the PI3K/Akt pathway, affords neuroprotection against an insult of sodium nitroprusside in human retinal pigment epithelial cells (Wang et al., 2015).

Interestingly, PI3K/Akt signaling controls its downstream effect or mTOR. PI3K/Akt/mTOR signals affect many functions, such as cell proliferation, growth, autophagy and apoptosis (Yu and Cui, 2016). When there are no extracellular stimuli, Akt is cytoplasmic and inactive (Alessi et al., 1996). Thereafter, upon activation by PI3K, phosphorylated Akt is enrolled to the plasma membrane (Alessi et al., 1997). Subsequently, Akt translocation facilitates phosphorylation of Thr308 on its activation loop (Alessi et al., 1996; Stokoe et al., 1997). To fully activate Akt, the rapamycin insensitive mTORC2 undergoes phosphorylation of Ser473 at its C terminal hydrophobic motif (Sarbassov et al., 2005). For mTORC1 activation, Akt suppresses TSC1/2, an inhibitor of Rheb1, thereby stimulating mTORC1 (Laplante and Sabatini, 2012).

In accordance with earlier studies showing that the dysfunction in the PI3K/AKT/mTOR pathway is manifested in diabetes (Bathina and Das, 2018) as well as many neurological diseases (Wang et al., 2017), our experiment indicated that activation of the PI3K/Akt/mTOR pathway was reduced in alloxan-treated mice, whereas treatment with CARB enhanced the PI3K/Akt/mTOR pathway. These data highlighted the beneficial effect of CARB that was, at least partly, linked to activation of the PI3K/Akt/mTOR pathway. Earlier studies by Ozdemir et al. (2014) proved that rapamycin affecting mTOR can suppress oxidative stress in diabetic retinopathy.

Furthermore, mTOR was found to participate in the attenuation of apoptosis by berberine in a diabetic retinopathy model in rats (Chen et al., 2018). This seems to run against the general consensus of inhibiting mTOR signaling pathway as a possible therapy for diabetic retinopathy. This consensus is based on reaching the vasculopathy stage of diabetic retinopathy. However, during the stage of neuropathy, mTOR signaling pathway could be offering an interesting target based on its neuroprotective effects, as depicted in many studies where PI3K/Akt/mTOR signaling activation affords neuroprotection. This is shown in traumatic brain injury rat model (Shen et al., 2017), in brain ischemia models (Lee et al., 2016) or in spinal cord injury (Li et al., 2019; Walker et al., 2019). This neuroprotective effect is regulated via its role in the balance between autophagy and apoptosis (Nguyen et al., 2010).

Neuronal loss is a key pathogenic mechanism for the development of diabetic retinopathy. Now, it is well-established that hyperglycemia and altered retinal metabolism caused progressive loss of neurons (Lynch and Abràmoff, 2017). Indeed, several studies reported increased c-caspase 3 activity in diabetic retinal neurons (Yang et al., 2013). To appraise the effect of CARB on retinal apoptosis, we assessed c-caspase 3 levels in diabetic retina sections. We found that CARB treatment attenuated retinal expression of c-caspase-3.

In conclusion, the results of the current work highlighted a protective role for CARB against retinopathy in diabetic mice. This beneficial effect is, at least in part, arbitrated by NGF/PI3K/Akt/mTOR activation. Future studies may be warranted toward using neuronal cultures to demonstrate the participation of the NGF/PI3K/Akt/mTOR pathway in neuronal survival induced by CARB. The mechanism by which CARB led to an increase in NGF level is a novel target to be identified for future drug development. Additionally, dosage optimization of CARB is necessary to avoid upregulation of NGF to harmful levels.

Based on the current results, treatment with CARB is expected to be useful in the neuropathy stages of diabetic retinopathy. More studies should be directed toward testing the effect of CARB in the vasculopathy stages of the disease. From a clinical point of view, future human studies are necessary to examine the action of CARB on diabetic retinopathy. Positive results may open avenue for a new convenient economically accepted oral treatment for diabetic retinopathy.

## Data Availability Statement

All datasets generated for this study are included in the article/Supplementary Material.

## Ethics Statement

The animal study was reviewed and approved by The Research Ethics Committee of the faculty of Pharmacy, Suez Canal University (No. 201603A2).

## Author Contributions

NE, YA-M, and SZ have contributed to the idea of the study. All authors have participated in the practical work, statistical analysis, graphic presentation as well as writing, proof-reading of the manuscript, and approval of the final form.

### Conflict of Interest

The authors declare that the research was conducted in the absence of any commercial or financial relationships that could be construed as a potential conflict of interest.

## Supplementary Material

Supplementary Images 1–9 Western blot whole run for total and phosphorylated TRKA, PI3K, Akt and mTOR where (i) is the saline group, (ii) Alloxan group, (iii) Alloxan+ CARB (25 mg/kg) and (iv) Alloxan+ CARB (50 mg/kg).
