# Supplementary figures and images for: Carbamazepine Alleviates Retinal and Optic Nerve Neural Degeneration in Diabetic Mice via Nerve Growth Factor-Induced PI3K/Akt/mTOR Activation
Source: Front Neurosci. 2019 Nov 1;13:1089. doi: 10.3389/fnins.2019.01089 (PMC6838003; doi:10.3389/fnins.2019.01089)

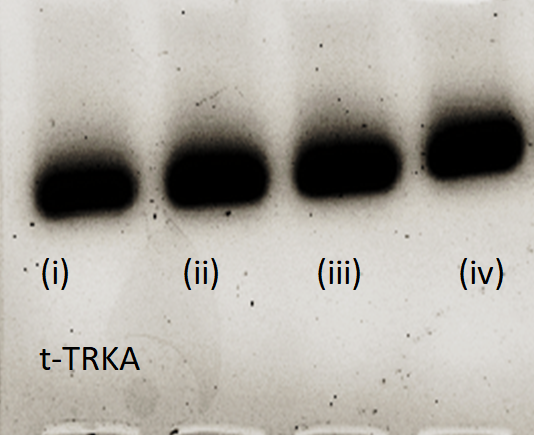

Supplement: Supplementary file 1 [file Image_1.tif]

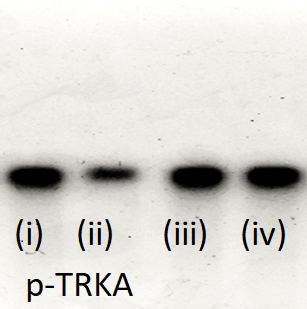

Supplement: Supplementary file 2 [file Image_2.tif]

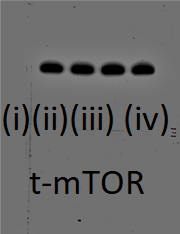

Supplement: Supplementary file 3 [file Image_3.tif]

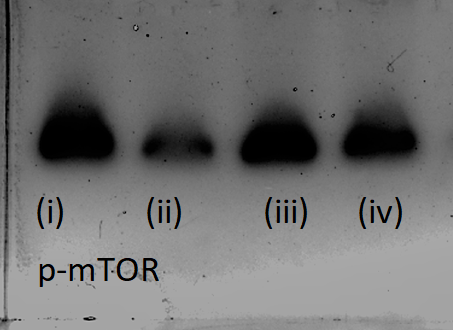

Supplement: Supplementary file 4 [file Image_4.tif]

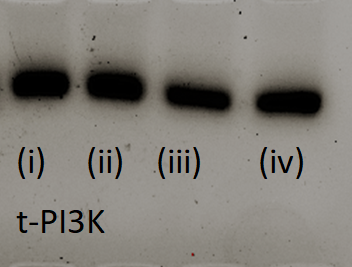

Supplement: Supplementary file 5 [file Image_5.tif]

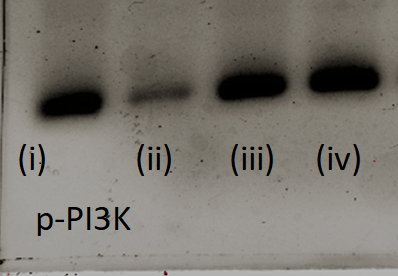

Supplement: Supplementary file 6 [file Image_6.tif]

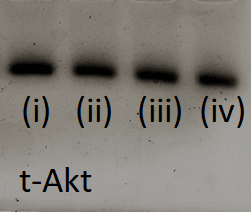

Supplement: Supplementary file 7 [file Image_7.tif]

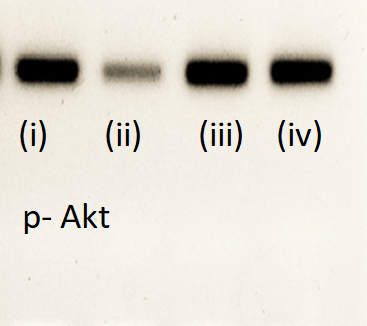

Supplement: Supplementary file 8 [file Image_8.tif]

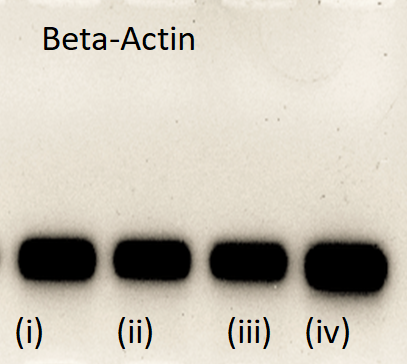

Supplement: Supplementary Images 1–9 — Western blot whole run for total and phosphorylated TRKA, PI3K, Akt and mTOR where (i) is the saline group, (ii) Alloxan group, (iii) Alloxan+ CARB (25 mg/kg) and (iv) Alloxan+ CARB (50 mg/kg). [file Image_9.tif]
